# *Rickettsia helvetica* in Patient with Meningitis, Sweden, 2006

**DOI:** 10.3201/eid1603.090184

**Published:** 2010-03

**Authors:** Kenneth Nilsson, Karin Elfving, Carl Påhlson

**Affiliations:** Uppsala University Hospital, Uppsala, Sweden (K. Nilsson, K. Elfving,); Mälardalens University, Eskilstuna, Sweden (C. Påhlson); Center of Clinical Research Dalarna, Falun, Sweden (K. Nilsson); Falu Hospital, Falun (K. Nilsson, K. Elfving)

**Keywords:** Rickettsia, PCR, isolation, meningitis, CNS, dispatch

## Abstract

Pathogenicity of *Rickettsia helvetica* is relatively unknown. We isolated a spotted fever group rickettsial organism from a patient with subacute meningitis. Nucleotide sequences of the 16S rRNA, *ompB*, and 17kDa genes identified the isolate as *R. helvetica*. This organism may be associated with serious infections such as central nervous system disorders.

*Rickettsia helvetica,* a member of the spotted fever group rickettsiae (SFGR), has been isolated from *Ixodes ricinus* ticks in many European and Asian countries. Although *I. ricinus* ticks are the main vector and natural reservoir, the organism has recently been found in *Dermacentor reticulates* ticks (*1*–*5*). Serosurveys have found antibodies reactive to *R. helvetica* in 1.9%–12.5% of the population in Lao People’s Democratic Republic, France, Italy, Denmark, and Sweden (*1*,*4*,*6*–*8*). The organism has mainly been considered nonpathogenic; several patients with a serologic diagnosis have had mild, self-limited disease with associated fever, headache, and myalgia. However, a more severe clinical disease has been demonstrated (*1*,*9*).

It is well known that Q fever and the rickettsial diseases typhus and spotted fever may cause central nervous system infection and that, of the SFGR, *R. rickettsii, R. conorii*, and *R. japonica* have a documented association with meningitis (*10*,*11*). We document a case of subacute meningitis caused by *R. helvetica*. The study was reviewed and approved by the Ethics Committee, Uppsala University, Sweden.

## The Case

In September 2006, a 56-year-old woman was hospitalized in Falun, Sweden, after 3 weeks of illness with gradually worsening headache and fever. She had no lymphadenopathy, rash, eschar, or history of tick bite or tick exposure. Radiographs showed a small, retrocardial pulmonary infiltrate, but the patient had few, if any, respiratory symptoms. Laboratory tests showed elevated C-reactive protein (56–128 mg/L), slightly elevated to reference-level leukocyte count (9,800–12,000 cells/μL), slightly low thrombocyte count (150,000 cells/μL), and reference values of hemoglobin and of aspartate and alanine aminotransferases. Cerebrospinal fluid (CSF) showed a slight pleocytosis (28 cells × 10^8^/L, of which 18 ×10^8^/L were mononuclear cells) but was otherwise within reference limits. CSF was stored at –20°C in a regular freezer and thawed only when used 1 year later. Negative results were obtained for blood and CSF cultures and for investigation for herpesviruses, tick-borne encephalitis, and enteroviruses. Urine was negative for *Legionella* and pneumococcal antigens, and serum was negative for antibodies against *Borrelia burgdorferi*. Computed tomography images of the brain and sinuses were unremarkable.

Intravenous administration of cefuroxime had no effect on the fever. Because atypical pneumonia was suspected, treatment was changed after 3 days to doxycycline (100 mg 2×/day). After 2–3 days the patient’s fever was gone, and she slowly recovered. The treatment was continued for 10 days. At a follow-up visit 1 year later, the patient was still well but had been asthenic for several months. No antibodies against *Mycoplasma pneumoniae* or *Coxiella burnetii* were found at the follow-up visit, and no other possible causative agent was confirmed. After giving informed consent, the patient was retrospectively included in an ongoing project that involved searching for fastidious organisms.

The patient’s previously frozen CSF was divided into 2 aliquots; bacterial DNA was extracted by using a MagNa Pure Kit (Roche Diagnostics GmbH, Mannheim, Germany) according to the manufacturer’s instructions. A genus-specific real-time PCR, as described by Stenos et al. (*12*), was used to detect SFGR. The PCR was performed in a Lightcycler 1.0 Real-Time PCR System (Roche Diagnostics GmbH) by using an LC Taqman Master Kit (Roche Diagnostics GmbH), primers, and TaqMan probe targeting the citrate synthase gene (*12*). To minimize risk for contamination, 0.25 µL LC uracil-DNA glycosylase (Roche Diagnostics GmbH) was included in each reaction. The positive control contained purified DNA of *R. helvetica* originally isolated from a domestic *I. ricinus* tick (*3*); the negative control contained sterile water. Positive samples were further analyzed by using 3 nested PCRs that amplify the 17kDa, outer membrane protein B (*ompB*), and 16S rRNA gene fragments as previously described (*3*,*13*,*14*) (Table). Amplification was conducted in a DNA thermal cycler (Hybaid, Ashford, UK) and a MJ Mini Gradient Thermal Cycler (Bio-Rad, Hercules, CA, USA), and expected fragment sizes were confirmed by gel electrophoresis in 2% agarose. Direct cycle sequencing analysis of both strands of nested PCR products was performed at the Center for Genomics and Bioinformatics, Karolinska Institutet, Stockholm, Sweden.

**Table:** Selected inner primers and probe used to amplify genes from cerebrospinal fluid of patient with subacute meningitis, Sweden, 2006*

Gene	Primers and probe	Nucleotide sequences, 5′ → 3′	Product size, bp	
*ompB*	*ompB*-IF	CCAATGGCAGGACTTAGCTACT	267	
	*ompB*-IR	AGGCTGGCTGATACACGGAGTAA		
17 kDa	RH 17-IF	GCATTACTTGGTTCTCAATTGG	214	
	RH 17-IR	AACCGTAATTGCCGTTATCCGG		
16SrDNA	Ric-F	TCTAGAACGAACGCTATCGGTAT	757	
	Ric-R	TTTCATCGTTTAACGGCGTGGACT		
*glt*A	SFG-CS-F	TGCCAAATGTTCACGGTACTTT	74	
	SFG-CS-R	CACAATGGAAAGAAATGCACGA		
	SFG-CS-Probe	TGCAATAGCAAGAACCGTAGGCTGGATG		

*ompB, outer membrane protein B; *gtlA*, citrate synthase.

Rickettsial DNA was amplified by real-time PCR from both CSF aliquots. Positive samples were further examined by using nested PCRs. The sequences obtained were 165 (17kDa) and 253 bp (*ompB*) and shared 100% similarity with the corresponding gene sequences of *R. helvetica* (GenBank accession nos. EU407139, EU407140).

To isolate the pathogen, we injected CSF from the frozen aliquot in volumes of 10 μL in a 25-cm^3^ flask into confluent monolayers of Vero cells and 80 μL in the other (*15*). After incubation, the cell culture was maintained in Dulbecco modified Eagle medium containing 10% fetal calf serum and kept in a humid cell chamber in 5% CO_2_, at 32°C, to allow rickettsiae to multiply. All cell lines and reagents were checked weekly for growth or bacterial contamination. Detection of growing rickettsiae was monitored by using Gimenez staining and an immunofluorescence assay of cells collected after centrifuging the medium and staining with rabbit antirickettsial hyperimmunserum and Alexa Fluor 488 goat antirabbit immunoglobulin (Ig) G (H+L) conjugate (Invitrogen, Carlsbad, CA, USA) as secondary antibody (Figure).

**Figure:**
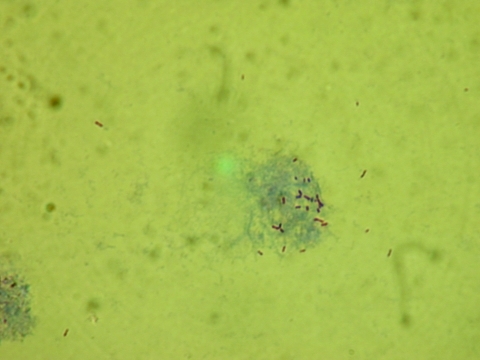
Rickettsiae in infected Vero cells. Sample from cerebrospinal fluid of patient with subacute meningitis, Sweden, 2006. Gimenez stain; original magnification ×1,000.

After 6 weeks, many intracellular bacteria were observed in the cells. Rickettsial DNA was verified by real-time PCR (*12*). The sequences obtained by nested PCR for the 17kDa and *ompB* genes in the isolate grown in Vero cells were identical to the sequences of the isolates obtained from the CSF (*3*,*13*,*14*). Amplification and partial sequencing of the 16S ribosomal RNA gene of the isolate produced fragments of 1,400 and 750 bp, respectively, which were 100% homologous to fragments of the deposited 16S ribosomal DNA sequence of *R. helvetica* from ticks (GenBank accession no. L36212).

SFGR antigen prepared from isolates grown in Vero cells of *R. helvetica* from an *I. ricinus* tick and from the patient was applied to each well of microscope slides. The antigen was dried, fixed in acetone, and incubated with serial dilutions of serum or CSF, as previously described (*7*). The positive control was serum from a patient with proven Mediterranean spotted fever and end-point IgG titers of 160 (provided by the Swedish Institute for Infectious Disease Control); the negative control was phosphate-buffered saline and serum from 3 healthy blood donors. IgG was detected by fluorescein isothiocyanate–conjugated γ-chain–specific polyclonal rabbit antihuman IgG (DakoCytomation A/S, Glostrup, Denmark). Microimmunofluorescence assay showed IgG end titers of 160 and 320 in the early-phase serum sample when the isolates of *R. helvetica* from tick and patient, respectively, were used as antigens. No antirickettsial IgG was detected in CSF when either isolate was used as antigen.

## Conclusions

For patients with fever and headache but no rash or eschar, diagnosis is difficult and can probably not be based only on epidemiologic, clinical, and standard laboratory criteria. It therefore seems that in SFGR-endemic areas, SFGR should routinely be included in the differential diagnosis of cause of meningitis. Appropriate antimicrobial drug therapy is essential for prompt recovery and prevention of complications.

SFGR isolation is usually not available in ordinary hospital laboratories and is too time-consuming to be a diagnostic alternative in clinical settings. Although this patient’s CSF had been stored in a regular freezer for 1 year, the rickettsial organisms were still viable.

PCR seems to be the most practical way to diagnose a suspected central nervous system disorder such as meningitis. The amplified nucleotide sequences were long enough to exclude other related rickettsial species. For example, the differences from other related rickettsiae were 10 and 5 nt for the *R. monacensis* 17kDa and *ompB* gene fragments, respectively, and 8 nt for *R. slovaca* *ompB*. Our study suggests that *R. helvetica* may cause infection of the CNS. When seeking to diagnose possible agents of meningitis, the usefulness of PCR and the relevance of the broader clinical spectrum of acute febrile illness caused by *R. helvetica* should be considered.
